# Oxidative stress and antioxidant status in primary bone and soft tissue sarcoma

**DOI:** 10.1186/1471-2407-11-382

**Published:** 2011-08-27

**Authors:** Fatima M Nathan, Vivek A Singh, Amreeta Dhanoa, Uma D Palanisamy

**Affiliations:** 1 Jeffrey Cheah School of Medicine and Health Sciences, Monash University Sunway, Jalan Lagoon Selatan, 46150, Bandar Sunway, Malaysia; 2 Department of Orthopaedic Surgery, University Malaya Medical Centre, Lembah Pantai, 50603, Kuala Lumpur, Malaysia

## Abstract

**Background:**

Oxidative stress is characterised by an increased level of reactive oxygen species (ROS) that disrupts the intracellular reduction-oxidation (redox) balance and has been implicated in various diseases including cancer. Malignant tumors of connective tissue or sarcomas account for approximately 1% of all cancer diagnoses in adults and around 15% of paediatric malignancies per annum. There exists no information on the alterations of oxidant/antioxidant status of sarcoma patients in literature. This study was aimed to determine the levels of oxidative stress and antioxidant defence in patients with primary bone and soft tissue sarcoma and to investigate if there exists any significant differences in these levels between both the sarcomas.

**Methods:**

The study cohort consisted of 94 subjects; 20 soft tissue sarcoma, 27 primary bone sarcoma and 47 healthy controls. Malondialdehyde (MDA) and protein carbonyls were determined to assess their oxidative stress levels while antioxidant status was evaluated using catalase (CAT), superoxide dismutase (SOD), thiols and trolox equivalent antioxidant capacity (TEAC).

**Results:**

Sarcoma patients showed significant increase in plasma and urinary MDA and serum protein carbonyl levels (p < 0.05) while significant decreases were noted in TEAC, thiols, CAT and SOD levels (p < 0.05). No significant difference in oxidative damage was noted between both the sarcomas (p > 0.05).

**Conclusions:**

In conclusion, an increase in oxidative stress and decrease in antioxidant status is observed in both primary bone and soft tissue sarcomas with a similar extent of damage. This study offers the basis for further work on whether the manipulation of redox balance in patients with sarcoma represents a useful approach in the design of future therapies for bone disease.

## Background

Sarcomas are malignant connective tissue tumours and can be classified into bone and soft tissue. While bone sarcoma tumours arise from the skeleton, soft tissue sarcomas are tumours of mesenchymal tissue such as blood vessels, fat and muscle [1]. Although soft tissue sarcomas account for less than 1% of malignant neoplasms, while bone sarcomas happen at a rate of one third of their soft tissue counterparts, a vast majority of these patients who develop these tumours eventually die from metastatic diseases [2].

Various factors contribute to the development of sarcomas that include environmental factors such as radiation, viral infection and chemical exposure. Heritable conditions also contribute to the occurrence of these tumours that include Li-Fraumeni syndrome, retinoblastoma and Werner's syndrome among others [3]. Various evidences exist supporting the role of oxidants in the development of cancers [4-6]. In bone tissues, recent studies have demonstrated ROS generation as a key modulator of bone cell function and that the pathophysiology of mineralised tissues is influenced by oxidative stress [5]. However, apart from the known risk factors, the function of oxidative stress in primary bone and soft tissue sarcomas remain to be explored further.

Reactive oxygen species (ROS) not only present as beneficial substances such as in chemotherapy and cancer apoptosis [3,4], but have also proven their role in carcinogenesis [3,7]. They are either formed via enzymatic reactions (respiratory chain, cytochrome P450 system and phagocytosis), or through non-enzymatic reactions such as those offset by ionising radiation and those involving oxygen with organic compounds [7]. The balance of ROS as a beneficial substance is accomplished by the antioxidant defence system that is composed of enzymatic (superoxide dismutase, SOD; glutathione peroxidase, GPx; glutathione reductase, GRx and catalase, CAT) and non-enzymatic (glutathione, GSH and coenzyme Q_10_, CoQ_10_) [4].

The imbalance between the pro-oxidants and antioxidants in favour towards the former gives rise to oxidative stress that has been proven to lead to carcinogenesis [4-6,8]. Increased ROS formation and decreased efficiency of the antioxidant defence not only causes the permanent alteration of biomolecular structures (DNA, proteins, lipids) but also their functions [4]. The damage done to these molecules are assessed based on the idea that although short-lived, ROS leave modified oxidative products hence, the presence of biomarkers. The autocatalytic process of oxidative destruction to polyunsaturated fatty acids (PUFA) caused by hydroxyl radicals and oxygen generates markers of lipid damage such as 4-hydroxynonenal (4-HNE) and malondialdehyde (MDA) [9]. Protein functions are altered via backbone fragmentation, side chain group oxidation, cross-linking and unfolding among others that give rise to markers of protein oxidation in human studies [10]. In cancer, it has been established that the higher the levels of oxidative stress, the more extensive the cancer [4]. In a study on paediatric acute lymphocytic leukaemia (ALL), children who acquired higher antioxidant levels at the onset of treatment had lesser complications and a better quality of life [8]. To date, findings on antioxidant defence and cancer imply that modifications to the antioxidant system can result in the alteration of ROS removal [6-8].

Our study aims to determine the role of oxidative stress in primary bone and soft tissue sarcoma patients by assessing lipid and protein damage as well as enzymatic and non-enzymatic antioxidant levels. This would provide a better understanding of the role of ROS in sarcomas that could lead to the development of new therapeutic strategies.

## Methods

The study consisted of 47 consecutive patients with bone and soft tissue sarcoma diagnosed at the Orthopaedic Oncology Unit, Department of Orthopaedics, University Malaya Medical Centre between 1 June to 31 December, 2009. These patients ranged from ages 7 - 78 years (28 males and 19 females). The diagnosis of sarcomas was based on clinical, radiological and histopathological findings. Representative samples of bone and soft tissue tumors measuring 1 × 1 centimeter were taken perioperatively during resection of tumour for histopathological diagnosis. All the patients were newly diagnosed primary bone and soft tissue sarcoma cases. The controls consisted of 47 healthy volunteers with ages and social conditions similar to those of patients. Consent was obtained from all patients and healthy individuals (controls) and the protocol had been approved by the Medical Ethics Committee of University Malaya Medical Centre (721.3/2009).

### Sample Collection

Blood samples were collected from 27 patients with bone sarcoma (ages 7 - 66 years) and 20 patients with soft tissue sarcoma (ages 8 - 76 years). Five mililiters of blood was collected from each patient; 1 mL for plain tube, 3 mL for ethylene diaminetetraacetate (EDTA) tube and 3 mL for citrated tube.

The blood collected in plain bottle without an anticoagulant system was centrifuged at 5,000 rpm for 10 minutes, the precipitate discarded and the serum obtained was utilised for protein carbonyl determination. Blood collected using EDTA as the anticoagulant was centrifuged for 10 minutes at 5,000 rpm and the resulting plasma was used in the determination of MDA, total thiol, SOD, CAT activities and trolox equivalent antioxidant capacity (TEAC). Urine obtained in urine specimen containers was used for MDA measurement. Samples acquired were stored at -80°C freezer until required for analysis.

### Measurement of Oxidative Damage

#### Determination of Lipid Peroxidation

Lipid peroxidation determination in plasma and urine was based on the formation of malondialdehyde-thiobarbituric acid (MDA-TBA) adduct by the reaction between MDA and TBA under acidic conditions at 100°C [11]. This was carried out using the Cayman's Thiobarbituric Acid Reactive Substances kit where absorbance of the samples was measured at 532 nm using the Bio-Rad Benchmark Plus Microplate Reader. The concentration of MDA was determined using an MDA standard curve. Normal human urine has a lipid peroxide level (expressed in terms of MDA) of 0.8-2 μmol/g creatinine [11]. Results were expressed as micromoles MDA per litre plasma and micromoles MDA per gram of creatinine for urine samples.

#### Determination of Protein Carbonyl

Oxidative damage to proteins was determined in the serum based on the formation of protein-hydrazone as a result of the reaction between 2,4-dinitrophenylhydrazine (DNPH) and protein carbonyls [12] using the Cayman's Protein Carbonyl Assay kit. Absorbance of the samples was measured at 370 nm using the Bio-Rad Benchmark Plus Microplate Reader. Carbonyl content was determined using the extinction coefficient of DNPH (0.022 μM^-1^cm^-1^). The total serum protein was then measured at 280 nm using Levine's method [13] on a LS-55 Fluorescence Spectrophotometer. Bovine Serum Albumin (BSA) was used to construct a protein standard curve. Protein carbonyl content in the serum was then expressed as nanomoles per milligram protein.

### Measurement of Antioxidant Status

#### Determination of Catalase Activity

The activity of catalase in the plasma using the CAT peroxidatic ability for enzyme activity determination where aliphatic alcohols function as specific CAT substrates to form an aldehyde [14] was analysed using the Cayman's Catalase Assay kit. The formaldehyde formed in the reaction between the enzyme and methanol in the presence of optimal hydrogen peroxide concentration was measured calorimetrically with 4-amino-3-hydrazino-5-mercapto-1,2,4-triazole (Purpald) [14]. The absorbance was measured at 540 nm on the Bio-Rad Benchmark Plus Microplate Reader and the reaction rate was determined using the formaldehyde standard curve. Results were expressed as unites per millilitre plasma. One unit was defined as the amount of enzyme causing the formation of 1.0 nmol formaldehyde per minute at 25°C.

#### Determination of Superoxide Dismutase Activity

SOD activity was measured in the plasma based on a tetrazolium salt to detect the formation of superoxide radicals by xanthine and hypoxanthine [15] using the Cayman's Superoxide Dismutase Assay kit. The absorbance was measured at 450 nm using Bio-Rad Benchmark Plus Microplate Reader. The SOD activity was measured using the linear regression equation from the standard curve. Results were expressed as units per millilitre plasma. One unit was defined as the amount of enzyme required to exhibit 50% superoxide radical dismutation.

#### Determination of Total Thiols

The determination of total thiols (T-SH) utilises an optimised enzymatic recycling technique using 5,5'-dithio-bis (2-nitrobenzoic acid) (DTNB) (CALBIOChem, USA) [16]. 20 μL of the diluted plasma sample was added into Eppendorf tubes followed by 400 μL methanol and 25 μL DTNB and the colour was left to develop for 20 minutes. The samples were then centrifuged at 3,000 × g for 10 minutes at 25°C. 90 μL was then loaded onto the 96-well microplate and the absorbance was measured at 412 nm using the Bio-Rad Benchmark Plus Microplate Reader. The T-SH concentration was calculated using the DTNB extinction coefficient (13.6 mM^-1^cm^-1^). Results were expressed as micromoles per litre plasma.

#### Determination of Trolox Equivalent Antioxidant Capacity

Plasma TEAC was determined by its ability to inhibit peroxidase-mediated formation of the 2,2'-azino-bis-3-ethylbenzthiozoline-6-sulfonate (ABTS^.+^) radical [17]. The capacity of the plasma antioxidant to inhibit ABTS oxidation was compared to the water-soluble vitamin E analogue (trolox) (Sigma-Aldrich Inc., USA). 20 μL of diluted samples were loaded onto respective wells on the 96-well microplate. 200 μL Chromagen (ABTS) (Sigma-Aldrich Inc., USA) was then added to these wells and the mixture left to react at 25°C for 6 minutes before reading the absorbance at 750 nm using the Bio-Rad Benchmark Plus Microplate Reader. The TEAC values were determined from the trolox standard curve. Results were expressed as millimoles per trolox equivalents per litre plasma. TEAC values were taken as the total antioxidant capacity in the plasma samples of the patients.

### Statistical Analysis

Triplicates of each sample were carried out in all of the above assays. SPSS Version 16.0 for Windows (Chicago, IL, USA) was used for data analysis. The significance of difference between the patients and controls as well as comparison between both sarcomas was determined using the Mann-Whitney U test (median values) as the data distribution was non-parametric. When data is non-parametric, it is suggested that median rather mean values be used. The independent t-test was used for parametric distribution. Probability values of p < 0.05 were considered significant.

## Results

### Oxidative Damage

Oxidative damage to lipid and protein biomolecules in the patients were measured by the level of lipid peroxides (MDA) formed in the plasma and the urine and protein carbonyl in their serum. The median values are as seen in Table 1.

**Table 1:** Oxidative stress levels by measurement of plasma and urine MDA and protein carbonyl of bone and soft tissue sarcoma patients and healthy individuals.

Individuals	n	Plasma MDA (μmol/L)	Urine MDA (μmol/g creatinine)	Protein carbonyl (nmol/mg protein)
**Sarcoma Patients**	47	7.30 ± 4.10	7.40 ± 10.90	0.79 ± 1.28

**Healthy Individuals**	47	2.40 ± 1.10	1.30 ± 0.70	0.37 ± 0.29

Values are median ± interquartile range

Mann-Whitney U test showed that significantly higher MDA levels were observed in the plasma and urine of sarcoma patients as compared to the controls. When measuring for protein damage, increase in protein carbonyl levels in the bone and soft tissue sarcoma patients were observed compared to controls, with a p-value of 0.000 (p < 0.05). Our results indicate a significant increase in both lipid and protein damage in patients with primary bone and soft tissue sarcoma.

### Antioxidant Status

The antioxidant status was assessed by studying the levels of their non-enzymatic (TEAC and total thiol) and enzymatic (SOD and CAT) antioxidants. Tables 2 and 3 show the median values of the respective parameters measured.

**Table 2:** Non-enzymatic plasma antioxidant status of bone and soft tissue sarcoma patients and healthy individuals.

Individuals	n	TEAC (mM)	Total SH (μmol/L)
**Sarcoma Patients**	47	0.64 ± 0.14	45 ± 8

**Healthy Individuals**	47	1.37 ± 0.06	144 ± 16

Values are median ± interquartile range

**Table 3:** Enzymatic antioxidant activity of plasma catalase and SOD of bone and soft tissue sarcoma patients and healthy individuals.

Individuals	n	CAT activity (U/mL)	SOD activity (U/mL)
**Sarcoma Patients**	47	15800 ± 2863	34 ± 34

**Healthy Individuals**	47	27800 ± 4314	154 ± 30

Values are median ± interquartile range

A lowered antioxidant defence system in sarcoma patients compared to the respective controls was noted (Table 2 and 3). TEAC was significantly lowered (53%) compared to the control group (p = 0.000, p < 0.05). Total thiol concentration as well was drastically lowered (69%) compared to the control group (p < 0.05) (Table 2).

It was also observed that the antioxidant enzymes, SOD and CAT, were significantly reduced in patients with bone and soft tissue sarcoma (p = 0.000) (Table 3). Catalase activity was decreased in sarcoma patients by 43% in comparison to the control value. A 78% decrease in SOD activity in sarcoma patients was observed as compared to the control. It was evident that the antioxidant enzymes (SOD and CAT) and non-enzymes (thiols and TEAC) measured in this study were significantly lowered in sarcoma patients compared to healthy individuals.

### Oxidative Damage in Bone and Soft Tissue Sarcoma

The extent of lipid and protein oxidation was compared between both the sarcomas. Table 4 shows that there is no significant difference between bone and soft tissue sarcoma in plasma (p = 0.081) and urinary (p = 0.998) MDA concentration and protein carbonyl levels (p = 0.085).

**Table 4:** Comparing oxidative damage in bone and soft tissue sarcoma by measuring plasma and urinary MDA and protein carbonyl content.

Sarcoma	n	Plasma MDA^a^ (μmol/L)	Urine MDA^b^ (μmol/g creatinine)	Protein Carbonyl^a ^(nmol/mg protein)
**Bone**	27	6.80 ± 3.10	9.50 ± 1.61	0.96 ± 2.02

**Soft Tissue**	20	8.20 ± 4.00	9.51 ± 1.39	0.71 ± 0.86

^a ^Median ± interquartile range

^b^Mean ± SE

### Antioxidant Status between Bone and Soft Tissue Sarcoma

**Figure 1:**
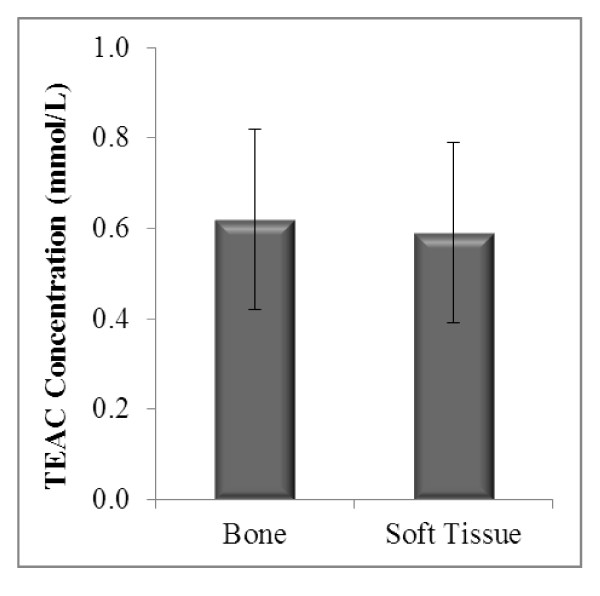
**TEAC concentration in bone and soft tissue sarcoma**.

**Figure 2:**
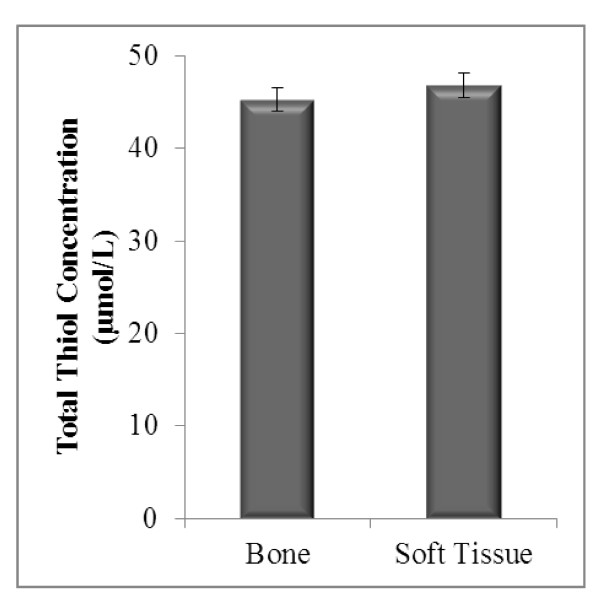
**Total thiol status in bone and soft tissue sarcoma**. Bone (*n *= 27) and soft tissue (*n *= 20). Results represent mean ± SEM. Independent t-test: no significant difference observed between the groups (*p *> 0.05).

The antioxidant status based on the levels of enzymatic antioxidants (SOD and CAT) and non-enzymatic antioxidants (TEAC and thiols) in both sarcomas were compared. TEAC concentrations in bone sarcoma were 5% higher (0.62 ± 0.03) than those with soft tissue sarcoma (0.59 ± 0.03), a difference which an independent t-test showed no significance (p > 0.05) (Figure 1). Total thiol concentration in bone (45.22 ± 1.26) was not significantly different from soft tissue sarcoma (46.80 ± 1.36) with a p-value of 0.406 (Figure 2).

**Figures 3:**
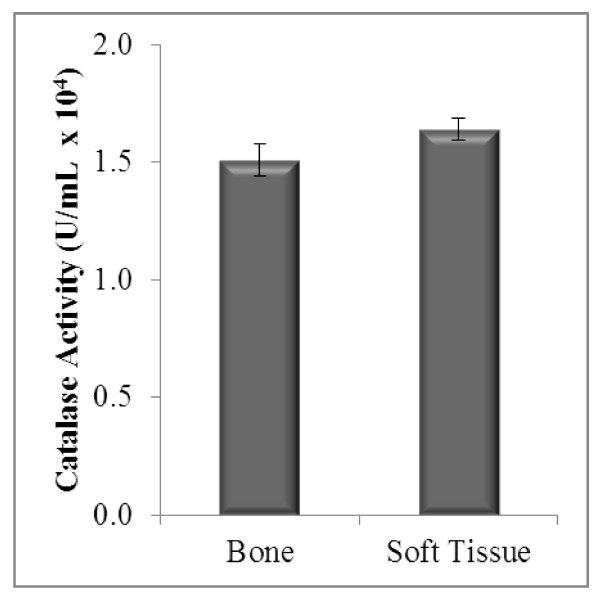
**Enzymatic antioxidant status (CAT activity) in bone and soft tissue sarcoma**.

**Figure 4:**
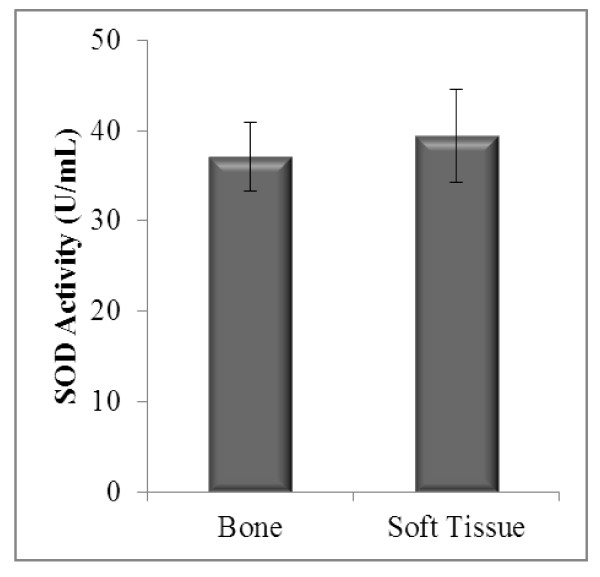
**Enzymatic antioxidant status (SOD activity) in bone and soft tissue sarcoma**. Bone (*n *= 27) and soft tissue (*n *= 20). Results represent mean ± SEM. Independent t-test: no significant difference between the groups (*p *> 0.05).

Catalase activity in bone and soft tissue sarcoma showed no significant difference (p = 0.146) (Figure 3). A difference of 5.81% in SOD activity between bone and soft tissue sarcoma patients was observed. This indicated a non-significant difference (p > 0.05) between the sarcomas. The mean SOD activities between both sarcomas are as shown in Figure 4.

## Discussion

This is the first study indicating the presence of oxidative stress and diminished antioxidant status in bone and soft tissue sarcoma. Free radicals attack lipids mainly PUFA, giving rise to lipid peroxides that play a pivotal role in cell division regulation [4]. The resulting MDA formed from lipid peroxidation functions as a tumour promoter and co-carcinogenic agent and has the ability to hinder the role of antioxidant enzymes. A direct correlation between lipid peroxidation and cell proliferation with increased lipid damage in highly proliferated cells has been noted [18]. In our study, urinary MDA levels were more pronounced than plasma MDA levels. While plasma MDA provides a picture of the MDA levels in the circulation of patients at the particular time blood is drawn, urinary MDA levels depict more of an end-product indicating the overall extent of lipid peroxidation. The levels expressed in urine provide a more accurate measure of lipid damage as aldehyde activity in urine is more concentrated [19]. Oxidative stress studies on various types of cancer have indicated increased MDA levels as compared to normal individuals [4,6,8]. These findings correlate with the levels of MDA measured in our study where a significant increase was observed in sarcoma patients as compared to healthy individuals.

Modifications to proteins are either elicited via direct oxidative attack (on lysine or arginine) or as a secondary reaction leading to the formation of protein carbonyls. However, studies are unable to differentiate between those directly produced by protein oxidation and those resulting from the addition of previously oxidised molecules [20]. Protein carbonyls are more advantageous as oxidative stress markers as oxidised proteins are generally more stable. They are produced in the circulation earlier with an elevation observed for at least four hours in the serum. Increased levels of protein carbonyl groups have been observed in various diseases [4,6,8]. Nevertheless, there is limited documentation related to protein oxidation in cancer [6]. The increase in protein carbonyls not only reflects oxidative stress but also protein dysfunction caused by the disease [21]. Similar observations were seen in our study where sarcoma patients showed significantly increased protein carbonyl levels as compared to the control group. Our findings of increased MDA and protein carbonyls imply the occurrence of oxidative stress in sarcoma patients as a result of lipid and protein damage.

The non-enzymatic antioxidant status (TEAC and total SH) as well as the enzymatic antioxidant status (CAT and SOD) was observed to have significantly decreased in the sarcoma patients. The diminished levels of antioxidant defence in the diseased individuals can be accounted for by two theories. In the first, circulating antioxidant reserves may have been exhausted in the attempt to counteract the DNA, lipid and protein damage. On another note, the elevated DNA, lipid and protein oxidation may have occurred as a result of a weakened defence system [22].

CAT functions in the conversion of hydrogen peroxide generated by a variety of reactions to water [23]. In bones, hydrogen peroxide has been demonstrated to oxidise proteins involved in cell differentiation and alter their activity by either causing inhibition or stimulation [5]. This suggests the possibility of hydrogen peroxide being the main ROS in bone tumour development. The possible raised levels of hydrogen peroxide may explain the reduction in CAT observed in bone sarcoma patients, in this study. Studies on colorectal cancer [6], urothelial bladder carcinoma [22] and ovarian cancer [24] have similarly demonstrated a decrease in both CAT and SOD. A reduction in CAT may also be accounted for by increased MDA forming cross-links hence, hindering the activity of membrane-bound enzymes [24].

A decrease in SOD activity was also observed in the sarcoma patients. Colorectal cancer [6] and other cancers [22,24] have shown similar reductions in SOD activity.

When subjected to moderate levels of oxidative stress, oxidation of cysteine residues has been found to cause mixed disulphide formations between protein thiol groups and low-molecular mass thiols (S-thiolation), mainly with GSH (S-glutathionylation). Protein function can be altered and regulated directly by protein S-glutathionylation that may also function in defence against irreversible oxidation. Under oxidative stress conditions these proteins accumulate, although they are readily reduced to free thiol groups when the normal redox balance is recovered by the glutaredoxins or reducing agents. A decrease in ratio of glutathione (GSH):glutathione disulphide (GSSG) (oxidised form:reduced form) indicates the occurrence of diseased states [25].

GSH plays a role in detoxification and bioreduction processes [4]. The decrease in thiol levels and enzymes involved in maintaining these levels (GPx) has been implicated in various types of cancer [4,22]. The decrease observed in these levels may result from its depletion due to the presence of increased hydrogen peroxide levels [4]. It was identified that decreased GPx activity may be a result of inactivation by the superoxide anion. With a reduction in GPx, the conversion of hydrogen peroxide to water remains incomplete [26]. GPx utilises GSH as a co-substrate in this defence mechanism [9] implying that an increase in hydrogen peroxide would cause more GPx to combine with GSH to produce GSSG. This process continues until the GPx and GSH reserves are exhausted accounting for a decrease in the total thiol levels. Our study on sarcoma patients showed a reduction in the total thiol levels.

Trolox, a water-soluble derivative of vitamin E was used to depict the antioxidant system [24]. It was shown that vitamin E present with lipids in the cell membrane inhibits cancer formation by neutralising ROS [4] and a direct relationship has been established with vitamin E deficiency and lipid peroxide production [27]. This evidence supports our findings where a decrease in plasma TEAC observed may be due to the increased lipid peroxidation observed. However, it should be noted that the low levels of vitamin E observed in this study may also be due to its hydrophobicity.

Sarcomas are defined as a type of cancerous connective tissue tumour [3]. As a result of different oxidative loads in varying tissues, the total antioxidant capacity varies. Individual tissues acquire their own antioxidant composition based on the oxidising courses that it most likely would endeavour [28]. Bone and soft tissue sarcomas arise from connective tissue [3], indicating the possibility that oxidising processes and antioxidant defence mechanisms in these two sarcomas are similar.

Tumours arising in bone and soft tissues share common characteristic features due not only to common mesenchymal origin but also the anatomical surrounding. Majority of bone sarcomas are bicompartmental during presentation, destroying the cortex and spreading directly into the soft tissue counterpart. On the other hand, soft tissue sarcomas are extracompartmental or found in an anatomical region that is not blocked off by anatomical barriers. These types of sarcomas extend only to adjacent compartments at later stages of the disease [3]. Regardless of the compartmentation, a clear link can be observed between sarcomas in the bone and soft tissue suggesting that the mechanism of oxidative damage and antioxidant defence would most likely be similar. This would explain the reason no significant difference in oxidative stress and antioxidant status in both sarcomas were observed.

## Conclusions

To summarise, our studies for the first time on oxidative stress and antioxidant status in both sarcomas clearly indicated an increase in oxidative stress (enhanced lipid and protein damage) and decrease in antioxidant status (lowered SOD, CAT, thiols, TEAC). These findings corroborate well with the oxidative stress and antioxidant status of patients suffering from prostate [29], colorectal [6] and ovarian [24] cancer. In addition, the similarities in oxidative damage and antioxidant defence between the bone and soft tissue sarcoma's suggests a possible link in the oxidising processes and antioxidant defence mechanisms in these two sarcomas. It is however noted that a larger cohort will provide a more significant result on age, gender and racial distribution in the individual types of bone and soft tissue sarcoma.

## Competing interests

The authors declare that they have no competing interests.

## Authors' contributions

FMN carried out the data acquisition, quality control and data algorithms, data analysis and interpretation, statistical analysis and participated in the preparation of the manuscript. VAS participated in the study concept, manuscript editing and final review. AD participated in the study concept, manuscript editing and final review. UDP conceived the concept and design, carried out quality control and data algorithms, data analysis and interpretation and participated in the preparation of the manuscript, manuscript editing and final review. All authors read and approved the final manuscript.

## Pre-publication history

The pre-publication history for this paper can be accessed here:

http://www.biomedcentral.com/1471-2407/11/382/prepub
